# COVID-19 Lockdown: Housing Built Environment’s Effects on Mental Health

**DOI:** 10.3390/ijerph17165973

**Published:** 2020-08-17

**Authors:** Andrea Amerio, Andrea Brambilla, Alessandro Morganti, Andrea Aguglia, Davide Bianchi, Francesca Santi, Luigi Costantini, Anna Odone, Alessandra Costanza, Carlo Signorelli, Gianluca Serafini, Mario Amore, Stefano Capolongo

**Affiliations:** 1 Department of Neuroscience, Rehabilitation, Ophthalmology, Genetics, Maternal and Child Health (DINOGMI), Section of Psychiatry, University of Genoa, 16132 Genoa, Italy; andrea.amerio@unige.it (A.A.); davidebianchi.md@gmail.com (D.B.); francesca.santi.ge@gmail.com (F.S.); gianluca.serafini@unige.it (G.S.); mario.amore@unige.it (M.A.); 2 IRCCS Ospedale Policlinico San Martino, 16132 Genoa, Italy; 3 Department of Psychiatry, Tufts University, Boston, MA 02111, USA; 4 Department of Architecture, Built environment and Construction engineering (DABC), Design & Health Lab, Politecnico di Milano, 20133 Milan, Italy; andrea1.brambilla@polimi.it (A.B.); alessandro.morganti@polimi.it (A.M.); stefano.capolongo@polimi.it (S.C.); 5 Department of Medicine and Surgery, University of Parma, 43121 Parma, Italy; luigi.costantini1@studenti.unipr.it; 6 School of Medicine, Vita-Salute San Raffaele University, 20132 Milan, Italy; odone.anna@hsr.it (A.O.); signorelli.carlo@hsr.it (C.S.); 7 Clinical Epidemiology and HTA, IRCCS San Raffaele Scientific Institute, 20132 Milan, Italy; 8 Department of Psychiatry, Faculty of Medicine, University of Geneva (UNIGE), 1206 Geneva, Switzerland; alessandra.costanza@unige.ch; 9 Department of Psychiatry, ASO Santi Antonio e Biagio e Cesare Arrigo Hospital, 15121 Alessandria, Italy

**Keywords:** COVID-19, lockdown, housing built environment, mental health, evidence-based design

## Abstract

Since the World Health Organization (WHO) declared the coronavirus infectious disease 2019 (COVID-19) outbreak a pandemic on 11 March, severe lockdown measures have been adopted by the Italian Government. For over two months of stay-at-home orders, houses became the only place where people slept, ate, worked, practiced sports, and socialized. As consolidated evidence exists on housing as a determinant of health, it is of great interest to explore the impact that COVID-19 response-related lockdown measures have had on mental health and well-being. We conducted a large web-based survey on 8177 students from a university institute in Milan, Northern Italy, one of the regions most heavily hit by the pandemic in Europe. As emerged from our analysis, poor housing is associated with increased risk of depressive symptoms during lockdown. In particular, living in apartments <60 m^2^ with poor views and scarce indoor quality is associated with, respectively, 1.31 (95% CI: 1046–1637), 1.368 (95% CI: 1166–1605), and 2.253 (95% CI: 1918–2647) times the risk of moderate–severe and severe depressive symptoms. Subjects reporting worsened working performance from home were over four times more likely to also report depression (OR = 4.28, 95% CI: 3713–4924). Housing design strategies should focus on larger and more livable living spaces facing green areas. We argue that a strengthened multi-interdisciplinary approach, involving urban planning, public mental health, environmental health, epidemiology, and sociology, is needed to investigate the effects of the built environment on mental health, so as to inform welfare and housing policies centered on population well-being.

## 1. Introduction

### 1.1. Lockdown Impact on Mental Health

Since the World Health Organization (WHO) declared the coronavirus infectious disease 2019 (COVID-19) outbreak a pandemic on 11 March [1], rapid and severe lockdown measures have been adopted by the Italian Government with school closures, border restrictions, quarantine of confirmed or suspected patients, and “stay-at-home” or confinement policies for all the residents [2,3]. For over two months of stay-at-home orders, houses became the only place where people slept, ate, worked, practiced sports, and socialized [4], accelerating the process of morphological changes of indoor ecosystems driven by technological evolution [5].

The potential benefits of mandatory mass quarantine need to be carefully weighed versus the possible impact on people’s daily life and negative psychological effects [6] compounded by the duration and difficulties of adhering to quarantine [7], fears of infection, frustration and boredom, inadequate supplies [8] and information, financial loss, and stigma, along with a daily physical activity decrease with consequences on non-communicable diseases (NCDs) [9,10].

As documented by a recent review, quarantined people are very likely to show mood lability, depressive and anxiety symptoms, irritability, insomnia, and acute and post-traumatic stress symptoms [11]. Severe depression, alcohol abuse, self-medication, and long-lasting avoidance behaviors have been reported as long-term effects (even up to three years after being quarantined). Moreover, along with social isolation and financial loss, quarantine would seem to increase suicidal ideation and behavior among at-risk populations [12,13].

### 1.2. Built Environment and Health

In recent decades, a growing number of studies have been conducted on the relationship between urban built environment and human health in both outdoor and indoor spaces [14,15,16,17]. The transactional nature of the relationship between subjects and place have been explored by several environment-behavior research studies and public health policies reflecting human ecology theory and applications [14,18]. Physical characteristics of the built environment, their affordances [19], and people’s individual characteristics are important to explore the association between built environment and health. Since the 1980s, socioecological theories have identified different built environment features as stress generators with an impact on mental health and individual performance that may be powerfully mitigated through environmental enhancements.

Socio-ecological approaches have been explored for integrating social, physical, cultural, and psychological aspects involving individuals–environment behavior. In particular, starting from Kaplans’ attention restoration theory [20] and Ulrich’s psychoevolutionary model [21], the effects of nature on human health have also been explored with regard to mental health. Grounding on the latter studies, evidence-based design (EBD) researchers studied the relationship between built environment characteristics and health and organizational outcomes in healthcare facilities, identifying architectural parameters that mostly impact occupants’ health or well-being [22,23,24]. Moreover, recent studies highlighted that interacting with natural environments [25,26] or just looking at them [17,27,28,29] may improve attention and reduce stress, with benefits for mental health and individual well-being. Although in the last few years, the debate around the potential role of the built environment on mental health flourished within the international scientific community, evidence from the literature is still scant, heterogeneous, mainly related to healthcare and working facilities, and frequently based on subjective well-being and small sample sizes [30,31,32].

To the best of our knowledge, this is the first large original study that investigates the effects of housing built environment on mental health during the COVID-19 lockdown.

## 2. Materials and Methods

### 2.1. Sample

A web-based survey questionnaire was sent by mail from 1 April 2020 to 1 May 2020 to students from a University Institute in Milan, Lombardy region, Italy. The study was performed three weeks after the COVID-19 epidemic outbreak in Italy. The total sample (*N* = 8177) consisted of undergraduate students, aged ≥ 18 years old who were invited to participate online, through a free Google Forms platform.

The survey was anonymous, and confidentiality of information was assured. Written consent was received from all individuals before participating in the questionnaire/study. Participants were allowed to terminate the survey at any time they desired, and no monetary rewards were given for completing the questionnaire.

### 2.2. Survey Questionnaire

The first section of the questionnaire investigated the general features of respondents: (a) gender, (b) current age, (c) marital status, (d) educational level in years, and (e) subjective impact of the mandatory confinement on working performance.

The second section consisted of the administration of the following evaluation scales that were designed to recognize depressive-, anxiety-, and sleep-related symptoms, impulsivity, and quality of life:

(a) The 9-item Patient Health Questionnaire (PHQ-9) [33] consists of nine items assessing depressive symptoms during the previous two weeks. The summed score ranges from 0 to 27, and the severity may be categorized into five categories: (1) normal (0–4), (2) mild (5–9), (3) moderate (10–14), (4) moderate–severe (15–19), and severe (20–27).
(b) The 7-item Generalized Anxiety Disorder scale (GAD-7) [34] consists of seven symptoms assessing anxiety symptoms during the last two weeks. Response options consisted of four answers: (1) “not at all”, (2) “several days”, (3) “more than half the days”, and (4) “nearly every day”, scored as 0, 1, 2, and 3, respectively. A total score ranging from 0 to 21 is possible by summing all items, and the severity can be categorized into four categories: (1) normal (0–4), mild (5–9), moderate (10–14), and severe (15–21).
(c) The 7-item Insomnia Severity Index (ISI) [35] assesses the severity of insomnia, categorized into four categories: (1) normal (0–7), (2) subthreshold (8–14), (3) moderate (15–21), and (4) severe (22–28).
(d) The Barratt Impulsiveness Scale–11 (BIS-11) [36] includes non-planning (a tendency to plan and think carelessly), attentional (refers to difficulties in focusing on a task and cognitive instability, such as racing thoughts and thought insertion), and motor impulsiveness (a tendency to act on the spur of the moment). Each item is rated on a four-point Likert scale from “never” to “almost always/always”, in which higher scores indicate higher levels of impulsivity.
(e) The Short Form 12-Item Health Survey (SF–12) [37] evaluates the health-related quality of life, including physical and mental component summary scores. The theoretical range varies from 0 to 100 with higher scores indicating better a quality of life.

The third section of the questionnaire investigated housing physical characteristics. Architectural parameters have been clustered into:

(a) Housing dimension in terms of net square meters;
(b) Presence/absence of a livable outdoor space (balcony or garden) measured in terms of balcony depth and garden property;
(c) Views typology (green or buildings) and subjective quality of views (poor or good/very good);
(d) Indoor quality defined by a set of parameters: natural lighting, acoustic comfort, thermo-hygrometric comfort, need for artificial lighting during the day, presence/absence of soft qualities in the living area such as art objects or greenery/plants, and presence/absence of privacy during phone calls for work or personal reasons. Furthermore, we considered the quality of indoor area high (6 to 7 satisfied parameters), medium (4 to 5 satisfied parameters), or poor (0 to 3 satisfied parameters).

### 2.3. Statistical Analysis

Statistical analysis was performed using the Statistical Package for Social Sciences (Version 25.0, SPSS; SPSS Inc., Chicago, IL, USA) for Windows, and the significance was set at *p* < 0.05 (two-tailed). Categorical variables were represented as count and percentage, while continuous variables were represented as mean and standard deviation (SD) considering sociodemographic and clinical characteristics.

The Kolmogorov–Smirnov test was performed to demonstrate the normal distribution of our sample. The sample was splitted into two subgroups according to the presence of a total score of PHQ-9 ≥ 15, which was the cut-off for the presence of moderate–severe and severe depressive symptoms. The Pearson χ^2^–test with Yates correction and t-test for independent samples were used to analyze the differences between two subgroups comparing categorical and continuous variables, respectively. 

Finally, a logistic regression analysis was used to explore the relationships between students with moderate–severe and severe depressive symptoms (dependent variable) and each of the other independent variables (architectural parameters) previously found to be associated in the statistical analysis, including gender and current age, as covariates. The probability of entering the equation was set at 0.05.

## 3. Results

Eight thousand one hundred seventy-seven students completed the survey, and the overall response rate (ORR) was around 31.5%. No questionnaire was returned incomplete. The male:female ratio was 1:1.003 with a current mean age and an educational level of 24.02 ± 7.46 and 14.74 ± 2.32 years, respectively. The most relevant sociodemographic and clinical characteristics are reported in Table 1.

Compared to students with absent to moderate depressive symptoms (PHQ-9 < 15), students with at least moderate–severe and severe depressive symptoms (*N* = 1050, 12.8%) showed a significantly higher severity for anxiety (13.56 ± 4.46 vs. 5.95 ± 4.01, *p* < 0.001), impulsiveness (63.66 ± 9.76 vs. 57.92 ± 8.18, *p* < 0.001), and sleep symptomatology (12.18 ± 5.77 vs. 5.83 ± 4.58, *p* < 0.001). Furthermore, a worse quality of life in both the mental (23.73 ± 6.06 vs. 39.28 ± 11.02, *p* < 0.001) and physical (47.08 ± 8.09 vs. 53.88 ± 5.25, *p* < 0.001) component summary was found significantly associated with the presence of moderate–severe and severe depressive symptoms. Additional statistical differences are summarized in Table 2.

With regard to the considered architectural parameters, students with moderate–severe and severe depressive symptoms significantly lived in apartments with small portioning (<60 m^2^) (13.3% vs. 7.3%, *p* < 0.001), with an unusable balcony (36.2% vs. 25.7%, *p* < 0.001), poor quality of indoor area (34.3% vs. 12.9%, *p* < 0.001) and a poor-quality view from the apartment (28.6% vs. 17.5%, *p* < 0.001). The other findings are displayed in Table 3.

When we performed a logistic regression analysis, students with PHQ-9 ≥ 15 were associated with apartment <60 m^2^ (odds ratio (OR) = 1.308), poor-quality view from apartment and indoor area (OR = 1.368 and OR = 2.253, respectively), and worsening of working performance (OR = 4.276) as shown in Table 4.

## 4. Discussion

Findings from our web-based cross-sectional survey indicated a worse quality of life with higher severity for anxiety, impulsiveness, and sleep symptomatology in students with at least moderate–severe and severe depressive symptoms. A strong association between poor housing and moderate–severe and severe depressive symptoms was found, with particular reference to small apartments, poor-quality, views and scarce indoor qualities. In addition, worsening working performance related to working from home increased the risk of depressive symptoms four-fold.

During infectious disease outbreaks, quarantine may be a necessary preventive measure. The quarantine’s potential benefits need to be carefully weighed versus the possible negative psychological effects.

As confirmed by recent studies [38,39], compared to non-quarantine subjects [40], quarantined individuals are significantly more likely to report psychological distress, anxiety, and depressive symptoms along with fear, irritability, anger, emotional exhaustion, and insomnia. Long-term behavioral changes after the quarantine period, such as vigilant handwashing and avoidance of crowds and the return to normality delayed by many months, have also been suggested [41].

Specific stressors may compound a negative individual psychological response either during (e.g., duration, fear about the own health or infecting others, boredom and frustration due to the loss of usual routine and confinement, insufficient clear guidelines about actions to take) or post-quarantine (e.g., financial loss, stigma) [10]. No data were published about the potential role of the housing built environment. However, given the previous available scientific evidence [30,42,43], some observations may be carried out.

Built environment includes human-made physical elements of the environment such as streets, open spaces, infrastructure, houses, and buildings, which could have an impact on the physical and mental health of the individual and health of a community [30].

A recent systematic review investigated the relationship between built environment and depressive symptoms [42]. Considering housing conditions, units with a poor housing quality and non-functioning or inadequate indoor facilities were related to current and lifetime depressive symptoms. Findings from our survey are in line with the results of the review. Small apartments without habitable balconies, with a poor housing quality such as a little natural lighting and acoustic comfort, a low thermo-hygrometric comfort, the absence of soft qualities in the living quarters (e.g., art objects, green plants), and living spaces, that do not guarantee adequate privacy during phone calls for work or personal reasons, were much more frequent in individuals with moderate–severe and severe depressive symptoms compared to those with absent to moderate depressive symptoms.

Views through a window influenced the mental health status of participants. We found a strong relationship between a poor-quality view from the apartment and moderate–severe and severe depressive symptoms. This is consistent with biophilia hypothesis [44], restoration theory [20], and the results of Ulrich’s studies in healthcare environments [10], as well as more recent literature reviews [11,29]. Viewing nature may elicit positive emotions, improve attention, reduce stress, and distract from focusing on pain [45,46]. Therefore, the more engrossing an environmental distraction is, the greater the pain reduction [47]. More recent studies confirmed the link between exposure to green space in the living environments and variations of stress levels, analyzing biomarker patterns such as cortisol secretion [25,28].

The impact of housing conditions on working performance during the COVID-19 lockdown was also investigated in our survey. The pandemic accelerated the pre-existing trend to work remotely, presenting a new set of challenges. Findings from our survey showed that depressive symptoms and poor housing quality affected working performance and made it worse. In particular, social isolation and living 24 h of the day in small apartments without a designated work-space available and with difficulties in defining work and leisure times may have led to decreased productivity. Although data on “home-office” configurations are not yet available, the strong association between perceived productivity and the physical configuration of corporate offices was recently confirmed by a recent study [48].

Finally, poor housing physical conditions may also impact physical health and health inequalities, and more detailed national and international regulations should be addressed in this direction to prevent even more enhanced impacts during possible future long-term “stay-at-home” periods [49,50,51].

### Limitations and Strengths

This survey needs to be interpreted while taking into account its several strengths and limitations. The main strengths of this survey are the large homogeneous sample size and the use of validated evidence-based psychiatric assessment tools. The major shortcomings of the present study are related to self-reporting questionnaires, as their reliability could be biased by under-reporting, under-estimating, and misunderstanding the issues. The cross-sectional study design does not allow inferences on the temporal relationship between the variables and only shows measures of associations; unfortunately, no information on mental health status before the COVID-19 outbreak was examined to determine the pandemic’s impact on university students. Moreover, the low response rate and the recruitment of students from a single university limited the generalizability of the results. Finally, housing physical characteristics have been investigated with structured but not validated questionnaire due to the scant evidence published in the existing literature, and several data such as incomes and length of work from home (days) were not considered.

## 5. Conclusions

To the best of our knowledge, this is the first large original study investigating the effects of housing built environment characteristics on mental health during the COVID-19 lockdown. Our findings reveal a strong association between poor housing and moderate–severe and severe depressive symptoms, with particular reference to living in apartments which are small and have a poor-quality view and indoor area. In addition, worsening working performance related to working from home increased the risk of depressive symptoms four-fold.

Built environment is a key determinant of health, the quality of which depends on the availability of resources, site location planning, and green spaces. As confirmed by our study, housing design strategies should be focused on larger and more livable living spaces facing green areas. An interdisciplinary approach involving urban planning, public mental health, environmental health, epidemiology, and sociology is needed to investigate the effects of the built environment on mental health outcome (e.g., well-being, psychological distress, depression), so as to inform welfare and housing policies centered on population well-being [52,53], especially in COVID-19 times [54,55].

## Figures and Tables

**Table 1:** Sociodemographic and clinical characteristic of the total sample included.

	Total Sample (*N* = 8177)
Gender (females), N (%)	4082 (49.9)
Current age, mean ± SD	22.02 ± 2.88
Marital Status, N (%)	
Single	7999 (97.8)
Married	174 (2.1)
Separated/divorced	4 (0.1)
Widowed	0 (0.0)
Educational level, mean ± SD	14.26 ± 1.68
Physical Component Summary-12, mean ± SD	53.01 ± 6.13
Mental Component Summary-12, mean ± SD	37.28 ± 11.73
Patient Health Questionnaire-9, mean ± SD	8.51 ± 5.08
General Anxiety Disorder-7, mean ± SD	6.93 ± 4.80
Insomnia Severity Index, mean ± SD	6.65 ± 5.20
Barratt Impulsiveness Scale, mean ± SD	58.66 ± 8.62
Attentional	15.84 ± 3.25
Motor	19.32 ± 3.56
Non-Planning	23.51 ± 4.40

**Table 2:** Comparison of clinical characteristics (anxiety, sleep, impulsivity and quality of life) according to the presence of moderate–severe depressive symptomatology into student subgroup.

Mean ± SD	PHQ–9 ≥ 15 (*N* = 1050)	PHQ–9 < 15 (*N* = 7127)	*t*/X^2^	*p*
Physical Component Summary–12	47.08 ± 8.09	53.88 ± 5.25	−36.141	<0.001
Mental Component Summary–12	23.73 ± 6.06	39.28 ± 11.02	−44.741	<0.001
General Anxiety Disorder–7	13.56 ± 4.46	5.95 ± 4.01	56.495	<0.001
Insomnia Severity Index	12.18 ± 5.77	5.83 ± 4.58	40.425	<0.001
Barratt Impulsiveness Scale	63.66 ± 9.76	57.92 ± 8.18	20.671	<0.001
Attentional	18.41 ± 3.40	15.46 ± 3.05	28.878	<0.001
Motor	20.38 ± 4.28	19.16 ± 3.42	10.462	<0.001
Non-Planning	24.87 ± 4.81	23.31 ± 4.30	10.816	<0.001

**Table 3:** Comparison of architectural parameters according to the presence of moderate–severe depressive symptomatology in the student subgroup.

N (%)	PHQ–9 ≥ 15 (*N* = 1050)	PHQ–9 < 15 (*N* = 7127)	*t*/X^2^	*p*
Apartment				
<60 m^2^	140 (13.3)	521 (7.3)		
61–120 m^2^	567 (54.0)	3658 (51.3)	59.537	<0.001
>120 m^2^	343 (32.7)	2948 (41.4)		
Balcony not livable	380 (36.2)	1833 (25.7)	50.837	<0.001
View from apartment				
Green	366 (34.9)	2938 (41.2)	15.404	<0.001
Buildings	684 (65.1)	4189 (58.8)		
Quality of view from apartment			72.950	<0.001
Poor	300 (28.6)	1248 (17.5)		
Good or very good	750 (71.4)	5879 (82.5)		
Worsening of working performance				
No/little	361 (34.4)	5171 (72.6)	609.425	<0.001
Much/Very much	689 (65.6)	1956 (27.4)		
Quality indoor area				
Poor	360 (34.3)	922 (12.9)	357.307	<0.001
Medium	446 (42.5)	3114 (43.7)		
High	244 (23.2)	3091 (43.4)		

**Table 4:** Relationship between potential explanatory variables and moderate–severe depressive symptomatology: results from the stepwise logistic regression analysis.

	T	E.S.	Wald	*p*	OR	95% CI for EXP
Gender	0.314	0.125	2.525	0.152	0.852	0.820–1.115
Age	0.050	0.085	0.752	0.352	0.975	0.888–1.075
Apartment < 60 m^2^	0.269	0.114	5.541	0.019	1.308	1.046–1.637
Balcony not usable	0.144	0.078	3.393	0.065	1.154	0.991–1.345
Green view	−0.058	0.074	0.603	0.437	0.944	0.816–1.092
Poor-quality view	0.313	0.081	14.822	<0.001	1.368	1.166–1.605
Worsening of working performance	1.453	0.072	406.758	<0.001	4.276	3.713–4.924
Poor-quality indoor area	0.812	0.082	97.585	<0.001	2.253	1.918–2.647
Constant	−3.028	0.120	638.781	<0.001	0.048	
